# Reverse-correlating mental representations of sex-typed bodies: the effect of number of trials on image quality

**DOI:** 10.3389/fpsyg.2013.00476

**Published:** 2013-07-30

**Authors:** David J. Lick, Colleen M. Carpinella, Mariana A. Preciado, Robert P. Spunt, Kerri L. Johnson

**Affiliations:** ^1^Department of Psychology, University of California Los AngelesLos Angeles, CA, USA; ^2^Department of Neuroscience, California Institute of TechnologyPasadena, CA, USA; ^3^Department of Communication Studies, University of California Los AngelesLos Angeles, CA, USA

**Keywords:** reverse-correlation, social perception, sex categorization, body shape, number of trials

## Abstract

Sex categorization is a critical process in social perception. While psychologists have long theorized that perceivers have distinct mental representations of men and women that help them to achieve efficient sex categorizations, researchers have only recently begun using reverse-correlation to visualize the content of these mental representations. The present research addresses two issues concerning this relatively new methodological tool. First, previous studies of reverse-correlation have focused almost exclusively on perceivers' mental representations of faces. Our study demonstrates that this technique can also be used to visualize mental representations of sex-typed bodies. Second, most studies of reverse-correlation have employed a relatively large number of trials (1000+) to capture perceivers' mental representations of a given category. Our study demonstrated that, at least for sex-typed representations of bodies, high quality reverse-correlation images can be obtained with as few as 100 trials. Overall, our findings enhance knowledge of reverse-correlation methodology in general and sex categorization in particular, providing new information for researchers interested in using this technique to understand the complex processes underlying social perception.

People readily separate others into discrete categories (e.g., male/female, Black/White, gay/straight; Allport, 1954; Taylor et al., 1978; Brewer, 1988), and these acts of categorization provide an efficient method of information processing that helps perceivers navigate an otherwise infinitely complex social world (Macrae and Bodenhausen, 2000). Of the many categorizations that perceivers make, biological sex (i.e., male/female) is among the most critical (Fiske and Neuberg, 1990; Stangor et al., 1992; Johnson et al., 2012). Indeed, event-related brain potentials readily discriminate male from female targets within 200 ms of visual exposure (Ito and Urland, 2003, 2005; Mouchetant-Rostaing and Giard, 2003), and sex categorizations often emerge before other important categorizations, including race (Stangor et al., 1992). One reason that sex categorizations occur with such remarkable efficiency is that they serve adaptive purposes, allowing perceivers to detect potential mates (Maner et al., 2007) and interpersonal threats (Johnson et al., 2012) with enough time to decide whether approach or avoidance is more prudent.

How do perceivers achieve such expedient sex categorizations? Prior research demonstrated that they utilize a variety of visually salient characteristics. For instance, sexually dimorphic cues in the face (Macrae and Martin, 2007; Freeman et al., 2008; Johnston et al., 2010) continuously and dynamically influence social perception to determine whether a person is categorized as male or female (Schyns et al., 2002; Freeman et al., 2008; Freeman and Ambady, 2011). Sexually dimorphic cues in the body also influence sex categorizations (Johnson and Tassinary, 2005; Lick et al., in press; Pollick et al., 2005; Aviezer et al., 2012). While these sex-typed bodily cues are numerous, two in particular have garnered recent empirical attention—body shape (waist-to-hip ratio; Johnson and Tassinary, 2005, 2007; Johnson et al., 2012) and body motion (gait pattern; Troje, 2002; Lick et al., in press; Pollick et al., 2005; Johnson and Tassinary, 2007). These cues help perceivers to accurately decode a person's sex in the early moments of person perception, but the precise ways in which perceivers expect men's and women's bodies to differ remain relatively unclear. In the current study, we explored reverse-correlation as a method for clarifying the bodily cues that perceivers use to categorize biological sex.

## The social perceptual interface: target features and perceiver knowledge

Although important, targets' visible features do not operate in isolation to determine sex categorizations; perceivers also bring pre-existing knowledge to the task of social perception. Indeed, a burgeoning literature in social vision has revealed that sex categorizations are biased by stereotype overlap with other social categories, including race (Johnson et al., 2012), sexual orientation (Johnson et al., 2007; Lick et al., in press), and emotion (Hess et al., 2009; Johnson et al., 2011). These biases reveal that perceivers' beliefs about social groups alter the sex categorizations they make (Freeman et al., 2012). Thus, the conceptual match—or lack thereof—between a target's features and a perceiver's knowledge appears to guide sex categorization.

A growing body of evidence supports our contention that perceivers match visible features of a stimulus to pre-existing knowledge structures in order to reach a categorization. According to prototype-matching theory, perceivers who encounter a novel stimulus endeavor to match it to a pre-existing category prototype (Rosch, 1973, 1998). Stimuli whose features match a prototype are categorized fluently, while those whose features do not match are categorized less fluently. Contemporary face perception models propose that a similar visual matching process underlies social categorization. Specifically, these models suggest that social categories are organized as “nodes” in a multidimensional space (Valentine and Endo, 1992; see also Corneille et al., 2007; Hugenberg et al., 2010). The nodes consist of densely organized clusters of individual exemplars (e.g., Mom, Grandma, Oprah Winfrey, Angelina Jolie) that characterize a category (e.g., female), and perceivers are presumed to categorize others by matching their features to an existing node. In support of this theory, researchers have shown that targets are classified more fluently when their features match the presumed mental representation of a given category (Medin and Schaffer, 1978; Cantor and Mischel, 1979; Basri, 1996; Hampton, 1998). Thus, both classic and contemporary theories contend that social categorization relies on the match between a target's visible features and a perceiver's pre-existing concept of a category.

## Reverse correlation as a tool for visualizing category knowledge

While theoretically compelling, it has been difficult to pinpoint the features that characterize perceivers' mental representations of a given category. Indeed, because these representations are mental constructs, researchers have traditionally relied on indirect methods (e.g., the efficiency with which perceivers disambiguate group membership given a pre-defined set of visual features) to draw inferences about their content. Recently, however, reverse-correlation has emerged as a data-driven method that enables researchers to visualize perceivers' mental representations of social categories (Todorov et al., 2011; Dotsch and Todorov, 2012). In a common reverse-correlation paradigm, perceivers identify the image from a pair that best depicts a particular category (e.g., female). In reality, the images are derived from an identical base image over which researchers have superimposed random noise. Over the course of many trials, the average of the chosen images is thought to approximate a perceiver's mental representation of the category in question. While the resulting images do not necessarily reveal the prototype for a category (Mangini and Biederman, 2004), they provide some indication of the salient features that perceivers use to identify members of that category.

Since its recent introduction as a method of visualizing mental representations of social categories, reverse-correlation has been used to probe perceivers' beliefs about the visual characteristics of many different groups. For example, researchers have used reverse-correlation to derive mental representations of sex categories (Mangini and Biederman, 2004; Nestor and Tarr, 2008; Johnson et al., 2012), sexual orientations (Dotsch et al., 2011), ethnic groups (Dotsch et al., 2011), emotions (Schyns et al., 2009; Jack et al., 2012), personality traits (Todorov et al., 2011), and even personal identities (Mangini and Biederman, 2004). In particularly striking demonstrations, researchers have extracted representations of happy emotions from patterns of pure noise (Gosselin and Schyns, 2003). This diverse and growing list of studies indicates that reverse-correlation is a powerful method for understanding the processes underlying social categorization.

Although reverse-correlation provides an elegant way for researchers to pinpoint the visual cues guiding social perception, the relatively limited use of this technique in sex categorization research has restricted our knowledge in at least two ways. First, previous research using reverse-correlation to understand sex categorization has focused almost exclusively on mental representations of faces. This early focus on the face is defensible, given that faces are among the richest sources of social information in one's environment, providing both individuating and categorical information (Hill et al., 1995; Farah et al., 1998; Zebrowitz and Montepare, 2008; Hugenberg et al., 2010). However, the communication of social identities is not restricted to faces. As noted above, there is a growing recognition that the body provides potent cues that inform sex categorizations (Johnson and Tassinary, 2005; Johnson et al., 2012). In fact, some have argued that body perception may be even more important than face perception, because it can occur at a distance that enables a perceiver to avoid unwanted interactions with another person (Zebrowitz and Collins, 1997; de Gelder, 2006; Sell et al., 2009). Despite the importance of this topic, we still have relatively limited information about perceivers' mental representations of men's and women's body shapes. One recent study provided initial evidence that that reverse-correlation may provide useful insights on this topic. Johnson et al. (2012) used reverse-correlation to determine whether perceivers hold extreme representations of men's and women's bodies. By obtaining objective measurements of waist-to-hip ratio from the classification images produced from a reverse-correlation task, they demonstrated that perceivers' mental representations of male and female bodies are indeed sexually dimorphic and quite extreme. However, it remains unclear whether perceivers' mental representations of human bodies reliably predict sex categorizations. Studies that test whether reverse-correlation images of men's and women's bodies are subjectively perceived to be highly gendered would help to clarify whether the differences in waist-to-hip ratio from Johnson et al. (2012) are perceptually meaningful to observers.

Second, previous research that has employed reverse-correlation as a tool to understand social categorization is limited because most studies have used large numbers of trials that may become untenable except among the most committed research participants. Indeed, many of the seminal studies in this area have exceeded 700 trials (e.g., Dotsch et al., 2011), with others employing as many as 2000 (Jack et al., 2012), 8000 (Smith et al., 2005), or even 20,000 trials (Nestor and Tarr, 2008). However, a growing number of researchers have begun using fewer reverse-correlation trials than their predecessors (e.g., 390 trials in Dotsch et al., 2008, Study 1; 640 trials in Karremans et al., 2011), which suggests a desire for more efficient methods. We are unaware of any published studies that have systematically examined how the number of classification trials affects the quality of the resulting images. While many factors may affect researchers' ability to obtain reliable content in reverse-correlation images (e.g., the base image, noise patterns, consensus in perceivers' mental representations of a given category), the ideal number of trials remains a crucial methodological question that will become increasingly important as researchers begin to employ reverse-correlation more widely. It is possible that the quality of classification images improves linearly as a function of the number of trials, but it is also possible that quality improves in a non-linear fashion, such that early trials achieve sufficient quality and additional trials provide relatively minor improvements. Understanding the association between number of trials and image quality will enable future researchers to maximize the efficiency of reverse-correlation protocols.

## The current research

Mindful of these limitations, we designed the current study with two aims—one conceptual and one methodological. First, we aimed to extend recent work by testing whether reverse-correlation methods provide reliably sex-typed body images that are perceptually meaningful to observers. In particular, we explored subjective perceptions of men's and women's bodies drawn from individual perceivers, offering new information about the validity of this technique for understanding sex categorization. Second, we aimed to provide the first systematic test of how the accrual of trials in reverse-correlation tasks affects the quality and clarity of the resulting classification images. Specifically, we examined the subjective quality of reverse-correlation images created with varying numbers of trials. Although our conclusions may be specific to mental representations of bodies derived using the specific methods described here, our approach will provide an empirically informed foundation and an analytic framework for future researchers to test the ideal number of reverse-correlation trials in their own domains of study.

## Methods

Our study involved two phases of data collection—(1) a *classification phase* during which participants completed a reverse-correlation task from which we derived their mental representations of sex-typed bodies, and (2) a *rating phase* during which a separate group of participants evaluated the images created during the classification phase in terms of their quality and sex typicality.

### Classification phase

In the classification phase, 36 undergraduates from the University of California, Los Angeles (23 women, 11 men, 2 unreported) participated in exchange for course credit or $10.

**Figure 1:**
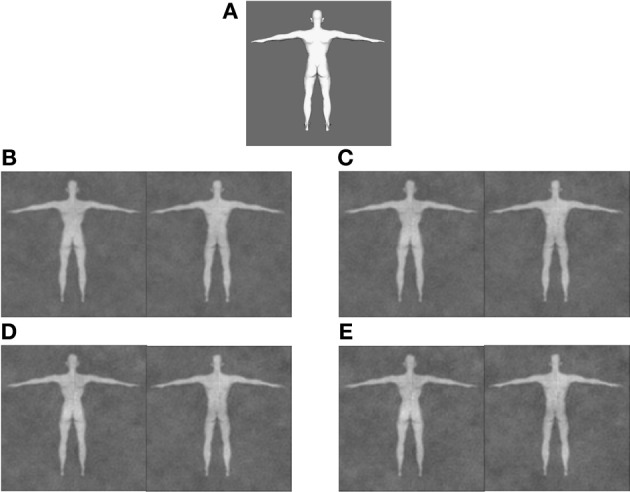
**Sample classification images including the original anthropometrically gender-neutral body stimulus (A), as well as group averages for female (left) and not-female (right) bodies derived from 100 trials (B), 300 trials (C), 500 trials (D), and 700 trials (E)**.

We began by creating an anthropometrically gender-neutral base image of a body facing backward with arms outstretched and legs in a wide stance [waist-to-hip ratio = 0.8049; 512 × 512 pixels; smoothed with a Gaussian filter at 10 × 10 pixels; root mean square (image contrast) = 0.1389; see Figure 1]. Then, using MATLAB (TheMathWorks, 2010) scripts from prior research (Dotsch et al., 2008), we created 700 pairs of images by adding or subtracting randomly generated noise (512 × 512 pixels) from the base. The noise patterns consisted of 60 sinusoids: 6 orientations (0°, 30°, 60°, 90°, 120°, and 150°) × 5 spatial scales (1, 2, 4, 8, and 16 sinusoid patches), each of which spanned 2 cycles per patch (0, π/2), with random contrasts. We weighted the noise patterns at 0.525 before superimposing them over the smoothed base image.

**Figure 2:**
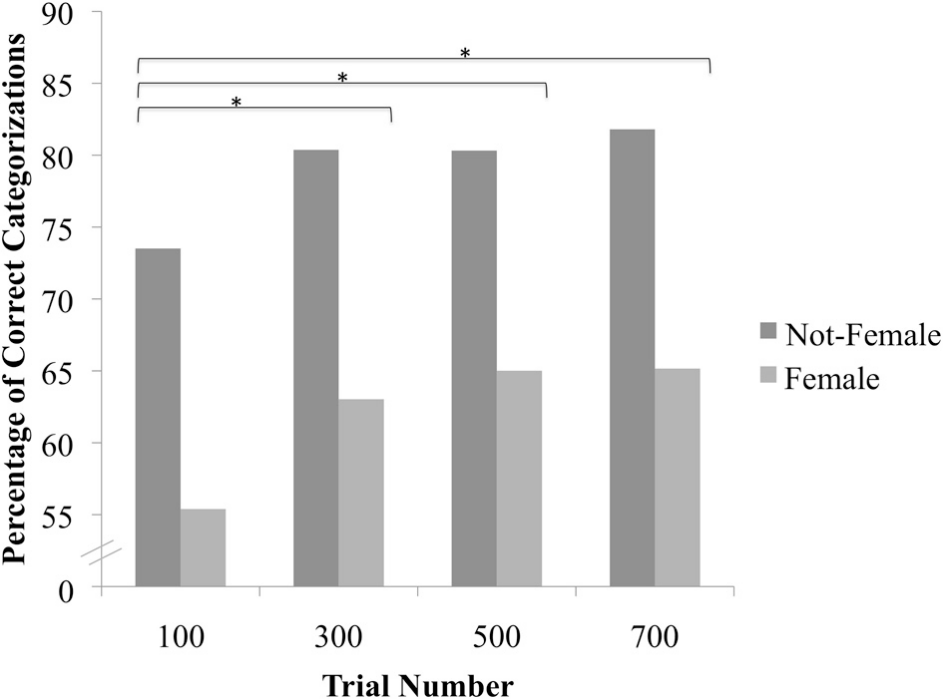
**Association between Classification Image Sex and Perceived Sex as a function of Trial Condition.** ^*^Indicates a significant contrast involving the effect of Classification Image Sex on Perceived Sex across trial conditions after applying the Sidak correction for multiple comparisons.

We used customized experimental software to present each stimulus pair side-by-side in a random order. In each trial, participants identified the image that best represented a woman's body using keys labeled *left* and *right* (see Figure 2). Most participants completed all 700 trials, though 2 stopped the study prior to completion. Of those who stopped prior to completion, one participant completed 572 trials and the other completed 672 trials.

At the end of the classification phase, we created composite female and not-female classification images for each participant by averaging the noise patterns of the selected and unselected images, respectively. Previous research has suggested that in some circumstances, classification images based on the unselected stimuli might represent the opposite of a binary social category (Dotsch and Todorov, 2012). For example, Johnson et al. (2012) found that the images *not* selected as female approximated male body shapes. Because sex is a binary trait, we inferred that images the current participants did not choose as representative of a female body were “not-female”—that is, morphologically male. While this assumption was reasonable given the binary nature of the category we explored (indeed, see results), it is important to recognize that the unselected images in a two-alternative forced-choice design may be less meaningful for continuous social dimensions (e.g., attractiveness, emotion).

We created multiple classification images for each participant, with separate female and not-female images derived from the first 100, 300, 500, and 700 completed trials. This resulted in eight total classification images per participant (288 images total; see Figure 1). For participants who did not complete all 700 trials, we used the maximum number of completed trials for the final category.

### Rating phase

In the rating phase, Internet users from Amazon Mechanical Turk evaluated the images created from the first 100, 300, 500, or 700 trials of the classification phase. We aimed to recruit 100 participants per trial condition for a total of 400 participants. Nine hundred eighty-six participants began the study; 369 completed it^1^. Participants who completed demographic questions were 30.46 years old on average (*SD* = 9.05), and they were diverse in terms of their sex (225 male, 139 female, 5 unreported), race (263 Asian, 76 White, 15 Biracial/Other, 10 Black, 4 Hispanic/Latino, 1 unreported), and sexual orientation (268 straight, 65 bisexual, 26 unsure, 8 lesbian/gay, 2 unreported).

After providing consent, participants were redirected to the survey-hosting website Qualtrics, where they were randomly assigned to evaluate one set of images (100-, 300-, 500-, or 700-trial images) produced during the Classification Phase. The images were presented individually at 512 × 512 pixels until participants rendered each judgment, which they made in three counterbalanced blocks. In the categorization block, participants provided basic social judgments of each image, including sex (*male, female*), gender (*1 = Extremely masculine* to *9 = Extremely feminine*), how confident they were in these judgments (*1 = Not at all confident* to *9 = Very confident*), how difficult it was to make these judgments (*1 = Not at all difficult* to *9 = Very difficult*), and how surprised they would be if the person in the image were actually of the opposite sex than they guessed (*1 = Not at all surprised* to *9 = Very surprised*). In the clarity block, participants rated the quality of each image across four 9-point scales (*1 = Not at all clear* to *9 = Very clear*; *1 = Not at all fuzzy* to *9 = Very fuzzy*; *1 = Not at all pixilated* to *9 = Very pixilated*; *1 = Not at all high definition* to *9 = Very high definition*). In the distinctiveness block, participants saw side-by-side pairs of female and not-female images created during the Classification Phase and rated how distinct they appeared (*1 = Not at all different* to *9 = Very different*) and how easy it was to tell them apart (*1 = Very difficult* to *9 = Very easy*). The images were presented in random order within each block, and the order of blocks was fully counterbalanced across participants. After the rating tasks, participants completed the importance subscale of the Gender Self-Esteem Scale (Luhtanen and Crocker, 1992), which assessed the centrality of gender to their self-concept across four items (e.g., “Being a man/woman is an important part of my self-image”). Finally, participants provided demographic information before being debriefed.

## Results

Our primary aims were: (1) to test whether reverse-correlation yields reliably sex-typed images of men's and women's body shapes, and (2) to explore associations between the number of trials used to create the images and subsequent image quality. Below, we address each of these aims in turn.

We created composite scales for the three items assessing Ease of Judgments (confidence, difficulty of categorization, surprise by incorrect categorization), the four items assessing Image Quality (clarity, fuzziness, pixilation, high-definition), and the two items assessing Distinctiveness (different, easy to tell apart). We assessed the reliability of each composite in two ways. First, we computed coefficient alpha from the residual variance/covariance matrix after accounting for random intercepts across both participants and stimuli for the variables in each composite. Using this method, alpha exceeded 0.61 for all scales. Second, we fit a one-factor model for each composite using a Bayesian estimator, again accounting for random intercepts across both participants and stimuli. Because these analyses were based on Bayesian estimates, we could not directly assess model fit, but we noted that all of the individual factor loadings were highly significant (*p* < 0.001), suggesting that the items in each composite contributed to a single latent factor. We also created a composite score for the gender identification scale (Luhtanen and Crocker, 1992). Because responses to this scale were not multilevel, we assessed reliability with traditional methods: Coefficient alpha was 0.53, and again, all of the items loaded significantly onto a single factor. Thus, all of our composite measures had modest reliability, perhaps because there were relatively few items in each scale. The fact that the items in each scale loaded onto a single latent factor provided rationale for using composite scores in our analyses.

Prior to conducting analyses, we effect-coded the categorical predictors including Classification Image Sex, Perceived Sex, Perceiver Sex, and Perceiver Sexual Orientation (−0.5 = *not female*, 0.5 = *female*; −0.5 = *male*, 0.5 = *female*; −0.5 = *straight*, 0.5 = *lesbian/gay/bisexual*), we dummy-coded Perceiver Race (White as reference category), and we coded Number of Trials as multi-categorical. We mean-centered continuous predictors (e.g., Perceived Gender—masculine/feminine, Perceiver Gender Identification, Perceiver Age).

Because participants provided multiple judgments of multiple stimuli, we tested our hypotheses using generalized estimating equations (Zeger and Liang, 1986), which are multilevel regression models that allow for accurate prediction of both dichotomous and continuous variables while accounting for within-subject dependencies in data. For all models, we report unstandardized regression coefficients and Wald *z*s. To test the robustness of our effects, we also tested models including Perceiver Age, Race, Sex, Sexual Orientation, and Gender Identification as covariates. The inclusion of these covariates did not change the pattern or significance of any result; therefore, we report the models without them. Instances in which removing incomplete responses affected significance levels are noted; all other results pertain to the full dataset, including participants with missing data.

### Reverse correlation and sex-typed body images

We first tested whether the reverse-correlation method yielded reliably sex-typed images of men's and women's bodies, regardless of the number of trials used to create the images. We approached this question in several ways. First, we sought to establish that perceivers' sex categorizations reflected the decision rules used to generate the classification images. To do so, we regressed Perceived Sex (male, female) onto Classification Image Sex (not-female, female), which revealed that perceivers categorized the bodies in the expected directions, *B* = 1.8322, *SE* = 0.0579, *z* = 31.63, *p* < 0.0001, OR = 6.2476. That is, perceivers tended to categorize female classification images as women (62.20% of the time) and not-female classification images as men (79.06% of the time). Intriguingly, perceivers were better at categorizing not-female bodies as male than categorizing female bodies as female. While not a primary focus of the current study, this finding replicates recent research demonstrating a marked male categorization bias in social perception (Johnson et al., 2012): In general, perceivers are more likely to categorize bodies as male than female, perhaps to avoid unwanted interactions with potential predators. This bias may have led to a higher rate of correct male categorizations in the current study.

In a parallel analysis, we regressed Perceived Gender onto Classification Image Sex. Again, perceivers judged target gender in the expected directions, *B* = 1.6869, *SE* = 0.0675, *z* = 25.01, *p* < 0.0001, rating female classification images as relatively feminine (*M* = 5.59, *SD* = 2.33) and not-female classification images as relatively masculine (*M* = 3.91, *SD* = 2.20) on a scale with a midpoint of 5. Finally, we explored the distinctiveness of each pair of female and not-female bodies. Mean ratings for Distinctiveness (*M* = 11.63, *SD* = 2.80) were significantly above the midpoint of the scale (i.e., 10), *t*_(564)_ = 9.5526, *p* < 0.0001, indicating that the female and not-female classification images were perceptually distinct.

Next, we employed a signal detection analysis (Stanislaw and Todorov, 1999) to test whether classification images provided sufficiently sex-typed visual information to afford perceptual sensitivity among observers. We coded correct female categorizations (i.e., categorizing a female classification image as female) as hits and correct male categorizations (i.e., categorizing a not-female classification image as male) as correct rejections, computing sensitivity (*d*′) with standard algorithms. Overall, *d*′ was significantly greater than 0 (*M* = 0.9113, *SD* = 0.7828), *t*_(604)_ = 28.6362, *p* < 0.0001, suggesting that the classification images contained sufficiently sex-typed bodily cues to compel accurate sex categorizations.

Collectively, these results indicate that the reverse-correlation technique used here yielded reliably sex-typed images of men's and women's body shapes. Based upon subjective ratings from independent perceivers, we found that female classification images were indeed categorized as female and perceived to be feminine. Not-female classification images were categorized as male and perceived to be masculine. Furthermore, perceivers rated pairs of female and not-female images as visually distinct. Thus, although our instructions prompted participants to identify the image that best depicted a woman with no mention of the category male, the unselected stimuli were reliably male-typed. Finally, a signal detection analysis revealed that the classification images provided sufficient visual cues to foster perceptual sensitivity in perceivers' sex categorizations. Although our findings cannot speak to absolute differences in the classification images of female and not-female bodies, in conjunction with recent data showing that mental images of men's and women's bodies differ objectively in waist-to-hip ratio (Johnson et al., 2012), they demonstrate that reverse-correlation yields perceptually meaningful and sexually differentiated images of men's and women's body shapes.

### Number of trials and classification image quality

We next tested whether and how the number of reverse-correlation trials affected the quality of the resulting classification images. Specifically, we explored each dependent variable as a function of Number of Trials, which we treated as four-level categorical variable (100, 300, 500, 700 trials). Because Number of Trials was a multi-categorical variable, we used Type 3 tests of fixed effects to determine the significance of all interactions. We first examined the sex typicality of classification images across trial conditions—that is, whether participants reliably differentiated the female from not-female images created with differing numbers of trials. To do so, we regressed Perceived Sex onto Classification Image Sex separately for each trial condition. Results indicated that participants reliably categorized image sex in the expected direction for all conditions (see Table 1 for regression parameters and odds ratios). That is, classification images were reliably sex-typed after as few as 100 trials, and they remained so for images created with 300, 500, and 700 trials.

**Table 1:** **GEE coefficients for regression of Perceived Sex onto Classification Image Sex for each trial condition**.

	***B***	***SE***	***z***	***OR***	***p***
100 Trials	1.2397	0.0996	12.44	3.4546	<0.0001
300 Trials	1.9496	0.1152	16.93	7.0259	<0.0001
500 Trials	2.0283	0.1296	15.65	7.6012	<0.0001
700 Trials	2.1385	0.1064	20.09	8.4867	<0.0001

We anticipated that the sex-typicality of the images might improve as the number of trials increased. To directly compare the sex-typicality of the images across conditions, we regressed Perceived Sex onto Number of Trials, Classification Image Sex, and their interaction. The two-way interaction was highly significant, *X*^2^(3) = 38.29, *p* < 0.0001. To decompose this interaction, we examined pairwise comparisons between all of the individual trial conditions after employing a Sidak correction (corrected α = 0.05/36 total comparisons = 0.0014). Results indicated that the association between Classification Image Sex and Perceived Sex (i.e., the tendency to rate female images as female and not-female images as male) was significantly stronger in the 300- (*B* = 0.7102, *SE* = 0.1522, *z* = 4.67, *p* < 0.0001), 500- (*B* = 0.7895, *SE* = 0.1635, *z* = 4.83, *p* < 0.0001), and 700-trial conditions (*B* = 0.8998, *SE* = 0.1458, *z* = 6.17, *p* < 0.0001) relative to the 100-trial condition; none of the other contrasts were statistically significant (Figure 2).

We also conducted a signal detection analysis to examine the extent to which each trial condition yielded classification images with visually compelling cues to the target's sex. As before, we coded correct female categorizations (i.e., categorizing a female classification image as female) as hits and correct male categorizations (i.e., categorizing a not-female classification image as male) as correct rejections to compute sensitivity (*d*′) with standard algorithms. We then subjected *d*' values to a one-way ANOVA with Number of Trials (100, 300, 500, 700) as a between-subjects factor. Results indicated that perceptual sensitivity varied significantly across trial conditions, *F*_(3, 601)_ = 8.8609, *p* < 0.0001, η^2^_p_ = 0.0424 (Table 2). Pairwise comparisons revealed higher sensitivity for classification images created with 300 trials (*B* = 0.3349, *SE* = 0.0909, *z* = 3.68, *p* = 0.0002), 500 trials (*B* = 0.3556, *SE* = 0.0963, *z* = 3.69, *p* = 0.0002), and 700 trials (*B* = 0.4045, *SE* = 0.0851, *z* = 4.76, *p* < 0.0001) relative to those created with 100 trials; none of the other contrasts were statistically significant.

**Table 2:** **Parameters for signal detection analyses**.

	**Hit (%)**	**Miss (%)**	**C.R. (%)**	**F.A. (%)**	***d*′**	***t***	***P***
100 Trials	55.3902	44.6098	73.5114	26.4886	0.6359	9.4855	<0.0001
300 Trials	63.0319	36.9681	80.3688	19.6312	0.9708	15.6944	<0.0001
500 Trials	65.0083	34.9917	80.3240	19.6790	0.9915	14.2423	<0.0001
700 Trials	65.1555	34.8445	81.7999	18.2001	1.0404	19.7117	<0.0001
Overall	62.2014	37.7986	70.0625	20.0938	0.9113	28.6362	<0.0001

**Figure 3:**
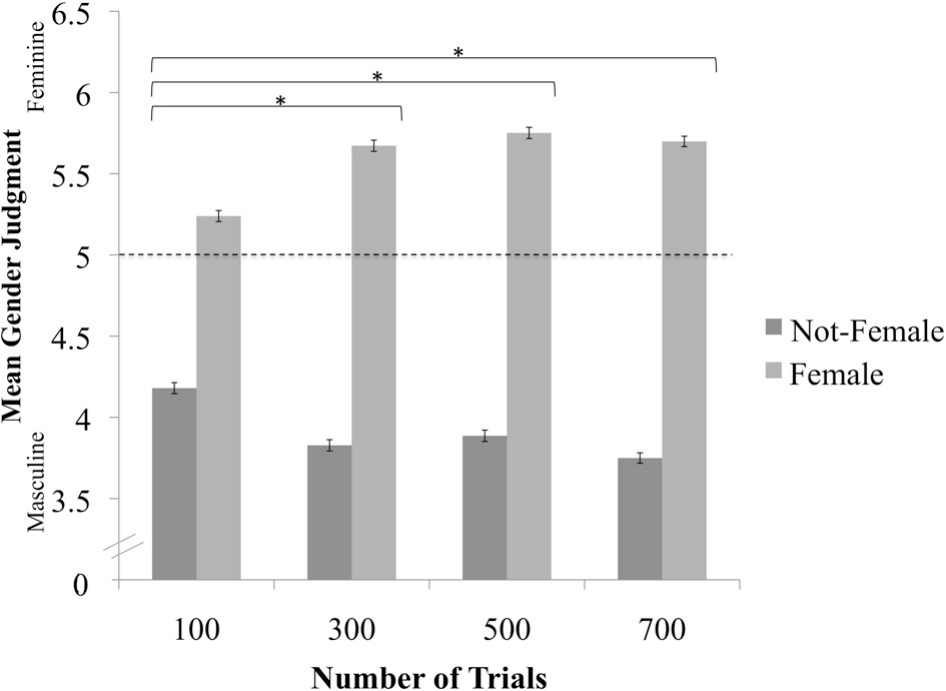
**Association between Classification Image Sex and Perceived Gender as a function of trial condition.** ^*^Indicates a significant difference after applying the Sidak correction for multiple comparisons.

Next, we regressed Perceived Gender onto Classification Image Sex separately in each trial condition. Similar to the results for Perceived Sex, participants judged gender in the expected direction (i.e., female bodies as feminine, not-female bodies as masculine) for all conditions (Table 3). These results provide further evidence that the classification images were reliably sex-typed after as few as 100 trials. To directly compare differences in the magnitude of this effect across condition, we regressed Perceived Gender onto Number of Trials, Classification Image Sex, and their interaction. Again, the two-way interaction was highly significant, *X*^2^(3) = 35.71, *p* < 0.0001. The association between Classification Image Sex and Perceived Gender (i.e., the tendency to rate female images as feminine and not-female images as masculine) was stronger in the 300- (*B* = 0.7879, *SE* = 0.1738, *z* = 4.53, *p* < 0.0001), 500- (*B* = 0.8093, *SE* = 0.1889, *z* = 4.28, *p* < 0.0001), and 700-trial conditions (*B* = 0.8987, *SE* = 0.1582, *z* = 5.68, *p* < 0.0001) than in the 100-trial condition; none of the other contrasts were statistically significant (Figure 3)^2^.

**Table 3:** **GEE coefficients for regression of Perceived Gender onto Classification Image Sex for each trial condition**.

	***B***	***SE***	***z***	***p***
100 Trials	1.0575	0.1028	10.29	<0.0001
300 Trials	1.8543	0.1402	13.16	<0.0001
500 Trials	1.8667	0.1585	11.78	<0.0001
700 Trials	1.9560	0.1202	16.27	<0.0001

**Figure 4:**
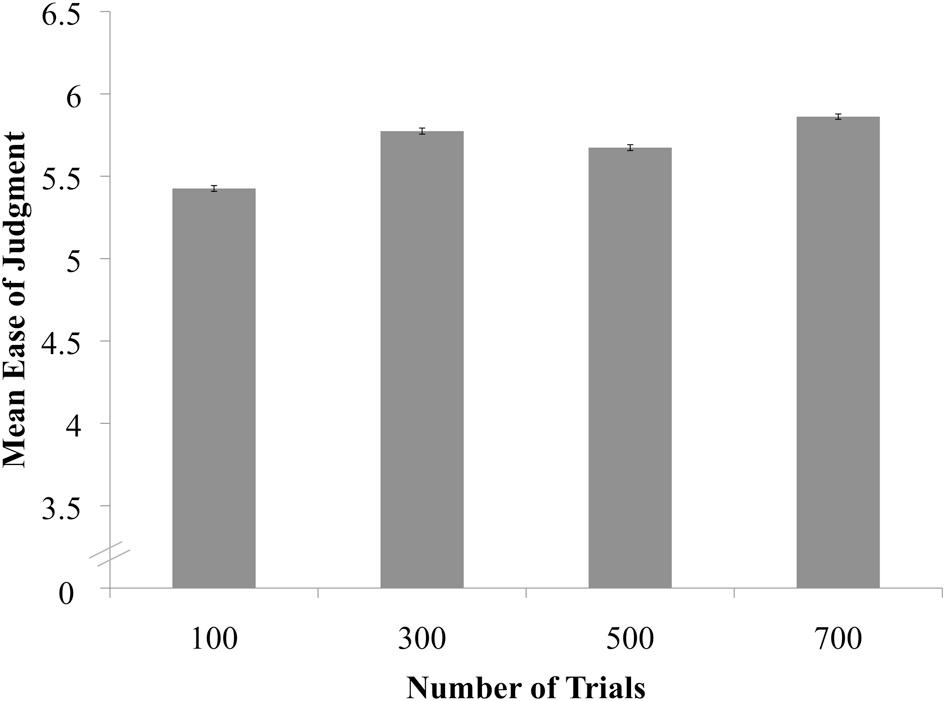
**Ease of Judgments for classification images as a function of trial condition**.

**Figure 5:**
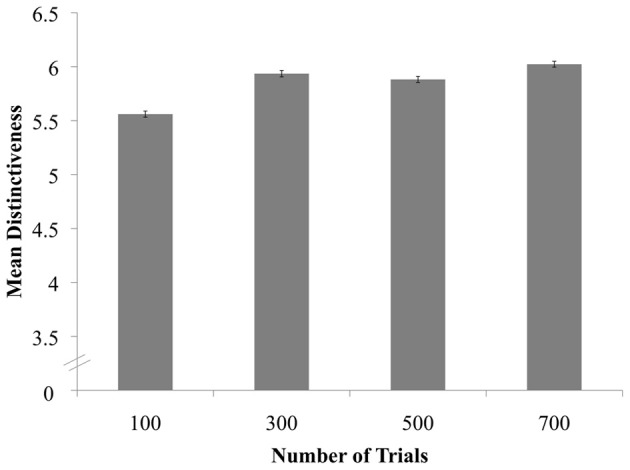
**Distinctiveness of female and not-female classification images as a function of trial condition**.

Next, we examined how image quality varied across trials. First, we regressed Ease of Judgments onto Number of Trials, which revealed a significant effect across conditions, *X*^2^(3) = 8.65, *p* = 0.0343^3^. Pairwise comparisons revealed that perceivers rated images created from 300 trials (*B* = 0.8159, *SE* = 0.3926, *z* = 2.08, *p* = 0.0377) and 700 trials (*B* = 1.1374, *SE* = 0.3914, *z* = 2.90, *p* = 0.0037) as easier to judge than images created from 100 trials, though these differences were not significant after the Sidak correction (Figure 4). We also regressed Clarity onto Number of Trials, revealing that Clarity ratings did not differ significantly across conditions, *X*^2^(3) = 5.31, *p* = 0.1507. Finally, we regressed Distinctiveness onto Number of Trials, revealing a significant effect of condition, *X*^2^(3) = 9.50, *p* = 0.0233. Perceivers rated images as more distinct in the 300- (*B* = 0.7662, *SE* = 0.3311, *z* = 2.31, *p* = 0.0206), 500- (*B* = 0.6489, *SE* = 0.3422, *z* = 1.90, *p* = 0.0579), and 700-trial conditions (*B* = 0.9071, *SE* = 0.3055, *z* = 2.97, *p* = 0.0030) relative to the 100-trial condition, though again, these differences were not significant after the Sidak correction (Figure 5)^4^.

In summary, reverse-correlation images of human bodies became reliably more sex-typed as the number of trials used to create the images increased. The images were also perceived to be of somewhat higher quality as the number of classification trials increased, though this trend was more evident for some measures of quality (e.g., perceived distinctiveness) than others (e.g., clarity). Importantly, the association between number of trials and image quality appeared to be non-linear: Our findings indicated a sharp increase in quality from 100 to 300 trials, but few notable improvements thereafter (see Figures 3–5). Thus, for mental images of men's and women's bodies created using the two-alternative forced-choice method described here, 300 trials may strike the ideal balance between participant effort and image quality.

## Discussion

Reverse-correlation has emerged as a powerful data-driven method for visualizing the cues that perceivers use to make social categorizations (Todorov et al., 2011; Dotsch and Todorov, 2012). The current study contributed two pieces of information to this growing literature. First, we found that reverse-correlation yields subjectively sex-typed images of men's and women's bodies, providing some of the first evidence that reverse-correlation is a valid method for assessing mental representations of human bodies. Second, we found that as few as 100 trials are sufficient to achieve high-quality images that reliably signal sex category information to naïve observers, though there were notable improvements in image quality from 100 to 300 trials.

Our finding that reverse-correlation images of men's and women's bodies were reliably sex-typed and categorically distinct have broad implications for understanding sex categorization. While most previous studies of social perception have used reverse-correlation to study perceivers' visual representations of faces, there is growing recognition that the body also provides important cues to sex category membership (Johnson and Tassinary, 2005; Johnson et al., 2012; Lick et al., in press). Our studies provide the first demonstration that body images derived from reverse-correlation techniques reliably signal biological sex to naïve observers. Indeed, perceivers in the current study tended to categorize female classification images as women and rate them as feminine, and they tended to categorize not-female classification images as men and rate them as masculine. Furthermore, perceivers rated pairs of female and not-female classification images as visually distinct from one another. While these findings do not pinpoint objective differences in these images, previous work from our lab showed that classification images of men and women derived from reverse-correlation methods vary consistently in their waist-to-hip ratio (Johnson et al., 2012). We suspect that mental representations of men's and women's bodies may also vary along other morphological dimensions, including frame size (i.e., women being physically smaller than men) and bicep size (i.e., women having smaller arms than men), and it would be useful for future researchers to explore these factors systematically. For now, our data indicate more generally that reverse-correlation is a useful method for understanding the bodily cues that perceivers use to make sex categorizations.

Furthermore, our finding that a relatively small number of trials yields high quality classification images of bodies has methodological implications for other researchers employing reverse-correlation techniques. While previous studies have found robust effects using a large number of reverse-correlation trials, it has remained unclear whether so many trials are necessary. Here, we found that classification images created from even the first 100 reverse-correlation trials were reliably sex-typed, and although image quality increased as the number of trials increased, this trend was not monotonic. Instead, we noted significant improvement in image quality from 100 trials to 300 trials, but relatively inconsequential improvements thereafter. These findings suggest that researchers may be able to use fewer trials than have been typical in previous reverse-correlation studies without compromising the quality or distinctiveness of the resulting images. In fact, at least for the bodily images created using the specific reverse-correlation methods described here, the number of trials necessary to derive maximally sex-typed images is certainly fewer than 700, and closer to 300.

While our findings suggest that reliable classification images can emerge from relatively few reverse-correlation trials, it is important to note that this parametric conclusion may only apply to sex-typed mental representations of bodies obtained using the specific technique described above. It remains possible that the number of trials required to obtain reliable classification images of faces differs from the number of trials required for bodies. Indeed, the sinusoid noise patterns used in this study may be especially effective at varying stimulus features with low spatial frequencies (e.g., bodies). These noise patterns might not be as effective at varying stimulus features at high spatial frequencies (e.g., faces), which suggests that more trials might be necessary to visualize those features. Furthermore, sex-typed body representations may be somewhat unidimensional, varying primarily in shape, while facial representations vary in myriad ways (shape, texture, pigmentation). More trials may be necessary to accurately model complex mental representations of human faces relative to bodies.

Other methodological considerations are also likely to affect the association between number of trials and classification image quality. For instance, we used pre-generated noise patterns to create a set of body images that were presented to all participants. Thus, the individual images that participants judged during the classification phase contained less variation than in some previously published work, so our findings may provide a conservative estimate of the number of trials necessary for obtaining high-quality mental representations of sex-typed bodies. Also, we used the unselected images in a two-alternative forced-choice design to derive not-female images. While previous research has suggested that the unselected images might approximate the opposite dimension of a binary category (e.g., not-female bodies approximate male body shapes; Johnson et al., 2012), and while participants in our study reliably categorized female images as female and not-female images as male, the number of trials necessary for reliably sex-typed classification images might vary if participants were to complete separate reverse-correlation tasks for male and female bodies. Collectively, these limitations lead us to caution researchers from inferring that 300 trials are ideal for all reverse-correlation paradigms. Nevertheless, the analytic strategy developed here is likely to provide a useful framework for others interested in examining the number of trials necessary to obtain reliable reverse-correlation images using different techniques (e.g., Gaussian white noise), stimuli (e.g., faces), and social categories (e.g., race).

In conclusion, people's tendency to efficiently process others on the basis of their social category memberships has inspired the development of novel reverse-correlation techniques to better understand the processes driving those categorizations. Until recently, however, most researchers have used reverse-correlation techniques to study perceivers' mental representations of faces. The current findings extend this method to bodies, demonstrating that as few as 100 trials provide a meaningful glimpse into the visual cues that characterize perceivers' beliefs about men's and women's body shapes. These insights will provide important foundations as reverse-correlation becomes a common method for studying the cues that people use to categorize others, especially with regard to their sex category memberships.

### Conflict of interest statement

The authors declare that the research was conducted in the absence of any commercial or financial relationships that could be construed as a potential conflict of interest.

## Footnotes

^1^Due to a coding error, our recruitment text indicated that the study would require 20 min when it actually required about an hour. Many participants elected to terminate the study after 20 min, which resulted in a high dropout rate. Because block order was fully counterbalanced, we assumed that responses were missing at random. Indeed, results were mostly unchanged when excluding participants with incomplete data. Instances in which excluding participants with incomplete data affected significance levels are noted, but all other results pertain to the full dataset.

^2^When removing Rating Phase participants with incomplete data and Classification Phase participants with fewer than 700 trials, the 100-trial and 500-trial conditions were no longer significantly different in gender-typicality after the Sidak correction (*p* = 0.0050). However, statistical trends were in the same direction as those reported.

^3^When removing Rating Phase participants with incomplete data and Classification Phase participants with fewer than 700 trials, Ease of Judgments varied marginally across conditions, *X*^2^(3) = 7.34, *p* = 0.0618, with no significant pairwise comparisons after the Sidak correction (*p*s > 0.01). However, statistical trends were in the same direction as those reported.

^4^When removing Rating Phase participants with incomplete data and Classification Phase participants with fewer than 700 trials, none of the Distinctiveness pairwise comparisons were significant after the Sidak correction (*p*s > 0.01). Statistical trends were in the same direction as those reported.
